# Correlation Between Type of Edentulism, Age, Socioeconomic Status and General Health

**DOI:** 10.3390/jcm14113924

**Published:** 2025-06-03

**Authors:** Simona Iacob, Radu Marcel Chisnoiu, Alina Zaharia, Mădălina Georgiana Bălaj, Adina Elena Iosa, Ana-Maria Condor, Andrea Chisnoiu, Smaranda Dana Buduru, Andreea Kui

**Affiliations:** 1 Department of Prosthodontics, Faculty of Dental Medicine, “Iuliu Hațieganu” University of Medicine and Pharmacy, 400012 Cluj-Napoca, Romania; iacob.simona@umfcluj.ro (S.I.); alina.zaharia@umf.cluj.ro (A.Z.); madalina.geor.balaj@elearn.umfcluj.ro (M.G.B.); adina.elen.iosa@elearn.umfcluj.ro (A.E.I.); ana.condor@umfcluj.ro (A.-M.C.); dana.buduru@umfcluj.ro (S.D.B.); gulie.andreea@umfcluj.ro (A.K.); 2 Department of Odontology, Endodontics and Oral Pathology, Faculty of Dental Medicine, “Iuliu Hațieganu” University of Medicine and Pharmacy, 400012 Cluj-Napoca, Romania; marcel.chisnoiu@umfcluj.ro

**Keywords:** edentulism, socioeconomic factors, general health, smoking, alcohol

## Abstract

**Background/Objectives**: Edentulism is a significant public health concern, particularly among aging populations, affecting oral functionality, aesthetics, and overall health. This study assessed the edentulism status of patients at the Prosthodontic Clinic of Cluj-Napoca, Romania, and explored the possible correlations with socioeconomic factors such as age, general health, smoking, and alcohol consumption. This study aimed to inform public health strategies to reduce edentulism incidence and improve overall oral health outcomes in Romania. **Methods**: The current study included 208 patients (127 females and 81 males). Each participant completed a standardized data collection form designed to gather comprehensive information on socio-demographic characteristics (including age, gender, and environmental origin), self-reported general health, and lifestyle habits related to smoking and alcohol consumption. The clinical examination was performed by the same operator, recording the odontal and periodontal status, as well as prosthodontic evaluation (including Kennedy class). **Results**: Findings indicated that female patients had more frequent class 3 and complete edentulism in the maxilla, while males predominantly presented class 3 in the maxilla and class 1 in the mandible. The age distribution revealed that patients aged 20–40 exhibited the highest prevalence of Kennedy class 3, while those over 60 showed a notable increase in complete edentulism (*p* < 0.05). Although most patients were from urban areas, no significant difference was found between origin and edentulism class. A significant link between alcoholism and mandibular edentulism was also identified (*p* < 0.05). **Conclusions**: Edentulism tends to progress with advancing age, often leading to more extensive tooth loss and the need for comprehensive dental rehabilitation. The condition is closely linked to general health status, highlighting its relevance as a potential indicator of systemic health risks. Lifestyle factors, particularly smoking and alcoholism, appear to contribute significantly to the deterioration of oral health, underscoring the importance of preventive strategies and early intervention.

## 1. Introduction

Edentulism remains a significant public health concern, particularly in aging populations. This condition not only affects oral functionality and aesthetics, but also has broader implications for general health and quality of life. Understanding the factors that contribute to edentulism is crucial for developing effective prevention and treatment strategies. Age is a well-established determinant of edentulism, with the risk of tooth loss increasing significantly as individuals grow older. This is often due to cumulative exposure to oral diseases and the natural aging of dental tissues [1].

A strong link exists between oral health and overall well-being, with dental conditions significantly influencing systemic health. Edentulism, a marker of poor prosthodontic status, is associated with a spectrum of systemic health issues, including malnutrition, cardiovascular diseases, and reduced quality of life. Furthermore, individuals with chronic diseases like diabetes and cardiovascular disease face an elevated risk of edentulism due to the systemic impact of these conditions on oral health [1,2,3]. Edentulism remains a prevalent issue, particularly among the elderly, with global estimates indicating that approximately 15–20% of adults aged 65 and older are edentulous, though prevalence rates can vary depending on socioeconomic and healthcare factors [4]. Lower socioeconomic status is strongly associated with higher rates of complete tooth loss, emphasizing the need for policies that address social determinants of oral health [5]. Governments and non-governmental agencies have dedicated considerable resources to oral health improvement in recent decades. Nevertheless, total tooth loss continues to be a leading contributor to disability-adjusted life years from oral diseases. Unfortunately, trends in periodontal health and tooth loss are not as thoroughly documented as those for dental caries [6].

The prevalence of prosthetic dental issues in Romania remains significantly high, with national and regional studies highlighting widespread issues such as untreated tooth loss, poorly fitted dentures, and limited access to prosthodontic treatment. A 2019 study reported that the prevalence of dental caries is 96.3% among young adults and 97.5% in the adult population, indicating a high burden of untreated dental conditions that may ultimately lead to edentulism and the need for prosthetic rehabilitation [7].

Despite the evident need for dental care, Romania records one of the lowest rates of dental service use in Europe. According to Eurostat data, the average number of dental consultations per individual was 0.5 times per year in 2017, far below the European average [8]. Financial constraints and geographical disparities exacerbate the problem, particularly in rural areas where access to dental services is severely limited. A 2023 study found that 90% of dental offices in Romania are private, with only a small fraction of costs being covered by state healthcare, leaving many individuals unable to afford necessary treatments [9].

Among the elderly population, the situation is particularly concerning. A study among institutionalized elderly individuals in the Bucharest area revealed that 64.3% of the oldest-old subgroup suffered from complete bimaxillary edentulism, further emphasizing the urgent need for improved prosthodontic care [10].

Lifestyle factors such as smoking and alcohol consumption further exacerbate the risk of tooth loss. Smoking, in particular, is a major risk factor for periodontal disease, which is a leading cause of tooth loss in adults. A 2021 Mendelian randomization study demonstrated a strong association between tobacco smoking and an increased risk of periodontitis [11]. Similarly, excessive alcohol consumption has been associated with poor oral hygiene practices and an increased risk of oral diseases, leading to tooth loss [12].

Beyond oral discomfort, edentulism significantly impacts general health. Missing teeth often cause difficulty eating, leading to nutritional deficiencies and digestive issues. Moreover, edentulism can contribute to social isolation and depression due to decreased self-esteem and impaired communication. A 2022 study published in *Clinical Oral Investigations* explored the association between tooth loss and cognitive decline. The researchers highlighted that edentulism could lead to impaired masticatory function, subsequently affecting nutritional intake and increasing the risk of cognitive impairments such as mild cognitive impairment and dementia [13]. Therefore, the necessity of prosthodontic treatment, including the provision of well-fitting dentures and other restorations, is critical not only for restoring oral function but also for improving overall health outcomes and quality of life.

This study primarily aimed to assess the edentulism status of patients attending the Prosthodontic Clinic of Cluj-Napoca, Romania. Additionally, it explored the correlation between the patients’ edentulism status and socioeconomic factors such as age, general health, smoking, and alcohol consumption. By understanding these relationships, this study also aimed to inform public health strategies regarding the reducing of the incidence of edentulism and improving overall oral health outcomes.

## 2. Materials and Methods

A cross-sectional observational study was performed for the assessment of the prevalence of edentulism and exploration of associations with various factors (age, socioeconomic status, general health, lifestyle habits) at a single point in time. The study population consisted of adult patients (age 20 and above) attending the Prosthodontic Clinic at “Iuliu Hațieganu” University of Medicine and Pharmacy, Cluj-Napoca, Romania between January 2023 and December 2024. This study was conducted in accordance with the Declaration of Helsinki and approved by the Ethics Committee) of Iuliu Hatieganu University of Medicine and Pharmacy with the reference number AVZ 8/6 January 2023.

The inclusion criteria in the study group were represented by the presence of at least one edentulous space in the maxillary or mandibular arch, the ability to provide informed consent and complete the study questionnaires, and sufficient cognitive function to understand the study procedures and answer questions accurately, as assessed by a brief cognitive screening tool.

The exclusion criteria were represented by significant communication barriers (e.g., severe language difficulties, cognitive impairments) that would prevent accurate data collection, refusal to participate in the study, patients with severe systemic conditions (e.g., uncontrolled diabetes, active cancer treatment) that, in the opinion of the treating clinician, would significantly influence their oral health status independent of the factors under investigation, and patients who had undergone major oral or maxillofacial surgery within the past 6 months that could impact their edentulism status.

The sampling method was consecutive, and all eligible patients attending the Prosthodontic Clinic during the specified study period (January 2023 and December 2024) were invited to participate. The recruitment continued until the target sample size was achieved (based on the sample size calculation).

After explaining the study’s purpose, procedures, risks, and benefits, written informed consent was obtained from each participant. Participants completed a structured questionnaire that included the following: socio-demographic information: age, gender, place of residence (urban/rural), history of systemic diseases (cardiovascular disease, digestive, diabetes, neurological disorders, osteoarticular conditions); self-reported general health status; and lifestyle habits: smoking and alcohol consumption.

A calibrated and trained dentist conducted a comprehensive oral examination, recording the following: the number of remaining teeth and Kennedy edentulism classification. Intra-examiner reliability was assessed by having the examiner re-evaluate a random sample of 10% of the patients.

All data were anonymized by assigning a unique identification number to each participant. All patients gave their informed consent regarding personal data management.

For the assessment of patients’ edentulism status, the Kennedy classification system was used, as follows [14]:

- Class I: Bilateral edentulous areas located posterior to the remaining natural teeth.
- Class II: A unilateral edentulous area situated posterior to the remaining natural teeth.
- Class III: A unilateral edentulous area with natural teeth present both anterior and posterior to it.
- Class IV: A single but bilateral edentulous area crossing the midline, located anterior to the remaining natural teeth.

The data obtained were centralized and statistically analyzed using SPSS—Statistical Package for the Social Sciences version 29.0 for Windows. Descriptive statistics (means, standard deviations, frequencies, percentages) were used to summarize the characteristics of the study population. Chi-square tests were used to examine associations between categorical variables (e.g., gender, age group, socioeconomic status, general health status, smoking status, alcohol consumption, Kennedy classification). Statistical significance was set at *p* < 0.05.

To determine an appropriate sample size, an a priori power analysis was conducted using G*Power 3.1.9.7 software. This analysis was performed to ensure adequate statistical power to detect meaningful associations while minimizing the risk of Type I and Type II errors. This study aimed to detect a small-to-medium effect size (w = 0.25), which is considered clinically relevant for this type of exploratory study. The power level was set at 80% (0.80), and a significance level of 0.05 was used. Based on these parameters, the a priori power analysis indicated an optimal sample size range of 207 to 243 participants; the final sample size achieved in this study was 208, which fell within this range.

## 3. Results

The current study included 208 patients: 127 females (61.1%) and 81 males (38.9%). Kennedy classifications of partial edentulism (I, II, III, IV) and complete edentulism were assessed.

Most of the female patients had class 3 and total edentulism in the maxilla and class 1 in the mandible, while the male patients more frequently had class 3 in the maxilla and class 1 in the mandible (Table 1 and Table 2).

The age of the patients was divided into three categories as follows: 58 patients (27.9%) were aged 20–40 years, 72 patients (34.6%) were between 41 and 60 years old, and 78 patients (37.5%) were over 60 years old (Table 3).

**Figure 1:**
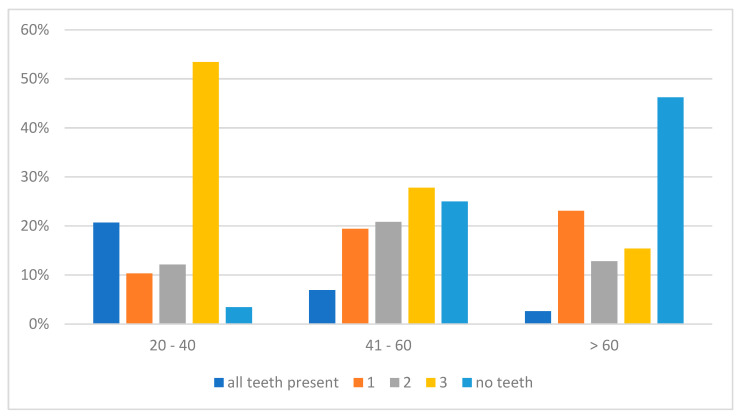
Prevalence of type of edentulism in the maxilla according to age groups.

**Figure 2:**
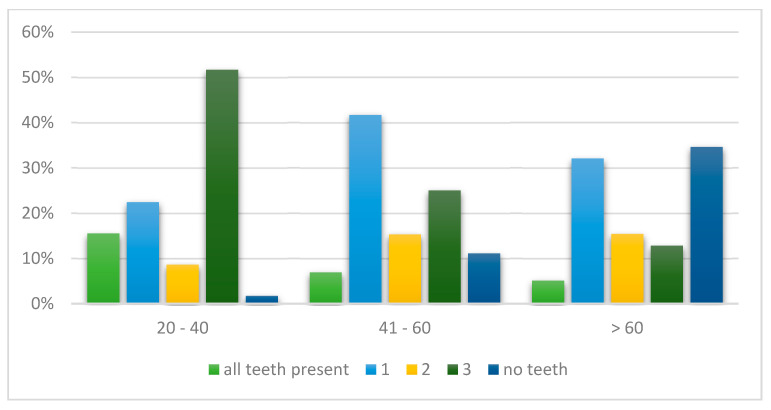
Prevalence of type of edentulism in the mandible according to age groups.

Regarding the prevalence of the type of edentulism, for both the maxilla and mandible in the age group 20–40, the highest number of cases with Kennedy class III were diagnosed, while in the age group 41–60 years old the most common class of edentulism was class III and complete edentulism in the maxilla and Kennedy class I in the mandible. For the patients over 60 years old, in the maxilla the highest rate of complete edentulism was observed, while in the mandible the most common were complete edentulism and class I (Figure 1 and Figure 2).

Additionally, patients completed a form about their general health to explore any potential link between specific health conditions and the type of edentulism in the maxilla and mandible. The results are displayed in Table 4 and Table 5. As expected, most patients were diagnosed with cardiovascular disorders, and among these individuals, most exhibited complete edentulism.

Most patients included in this study were from urban areas. Additionally, the chi-square test indicated no statistically significant difference between patients’ origin and the class of edentulism.

It is well established that alcoholism and smoking can affect oral health, so their correlation with the type of edentulism in patients was analyzed. A statistically significant relation was identified between alcoholism and mandibular edentulism (*p* = 0.029). The most common type of mandibular edentulism among alcohol consumers was class 1, while in the maxilla it was class 3. Among smokers, total edentulism was most common in the maxilla, and class 1 was frequently diagnosed in the mandible.

## 4. Discussion

The prevalence of edentulism continues to pose substantial challenges within public health, especially given its strong association with aging populations and socioeconomic disparities. The impact of tooth loss extends beyond oral function, influencing nutritional intake, psychological well-being, and overall health.

In this study, 208 patients were included, the majority of whom were females. This could be because women generally exhibit greater health awareness and are more proactive in seeking preventive care. This behavior extends to oral health, where regular dental check-ups are often prioritized to maintain good hygiene and detect potential issues early on. Women are also more likely to be concerned with dental aesthetics, such as the appearance of their smile [15].

The current study did not identify any statistically significant difference between gender and type of edentulism. This finding aligns with the conclusions drawn in the literature review by Jeyapalan and Krishnan [16], which analyzed multiple studies and reported no significant gender correlation with the occurrence of partial edentulism. However, the review also noted that some studies have observed significant relationships between gender and various classes of partial edentulism, with women often demonstrating greater awareness and a higher propensity to seek restoration for missing teeth, possibly due to increased concern about appearance and better health-seeking behavior.

With respect to the patients’ age and type of edentulism, the results of the current study indicate that in the younger group, class III edentulism was the most common. As age increases, the number of missing teeth rises, with edentulism shifting toward the posterior area of the dental arch. These observations are consistent with several previous studies. For instance, research by Jeyapalan and Krishnan [16] reported that younger adults are more likely to present with class III and IV removable partial dentures (RPDs), whereas older adults tend to have more distal extension RPDs, corresponding to class I and II edentulism. Similarly, a study conducted in Riyadh, Saudi Arabia, found that complete edentulism was predominantly observed in the elderly population, reinforcing the association between increased age and extensive tooth loss [17]. Furthermore, the study by Zaigham and Muneer [18] also reported that class III edentulism was the most common pattern in younger age groups. As age increased, there was a significant shift towards class I and class II edentulous arches, further supporting the correlation between age and the progression of edentulism.

The type of edentulism is strongly connected to general health, as the extent of edentulous spaces can significantly affect physical, psychological, and nutritional well-being. Individuals with severe edentulism, particularly those with complete tooth loss, often experience impaired chewing and digestion, leading to poor dietary intake and nutritional deficiencies. For instance, those who are edentulous may find it difficult to consume fiber-rich foods, fruits, and vegetables, which reduces their intake of vital nutrients such as vitamins, iron, and calcium. A 2024 overview of systematic reviews concluded that individuals with partial or complete tooth loss are more likely to be malnourished or at risk of malnutrition. This compromised nutritional status can lead to various health disorders, including anemia and osteoporosis, due to inadequate intake of essential nutrients such as iron and calcium [19]. These nutritional challenges could exacerbate cardiovascular diseases, diabetes, and dementia. Furthermore, the connection between edentulism and systemic diseases, including cardiovascular disease and diabetes, is well established. Chapple et al. [20] reported that individuals with advanced periodontal disease and tooth loss, especially class 1 or complete edentulism, were more prone to developing cardiovascular diseases as a complication of diabetes. The results of the current study also demonstrated a link between cardiovascular disorders and the type of edentulism in the mandible, where class 1 and complete edentulism were the most prevalent clinical situations.

The results of the current study showed no statistically significant difference between the type of edentulism and rural or urban origin. On the other hand, other studies have shown that individuals living in rural areas are more likely to experience higher rates and more severe forms of edentulism compared to those in urban settings [21]. This disparity is often linked to limited access to dental services, lower income, and less emphasis on preventive dental care in rural populations. For instance, a study by Luo et al. [21] reported that rural adults were less likely to receive preventive dental procedures compared to their urban counterparts. This disparity was attributed to factors such as limited access to dental services, lower socioeconomic status, and less emphasis on preventive care in rural areas. The discrepancy between the current study’s findings and those of Luo et al. may be influenced by differences in study design, sample populations, or regional factors. Additionally, variations in healthcare infrastructure, cultural attitudes toward dental care, and socioeconomic factors could contribute to these differing outcomes. These inconsistencies underscore the need for further research to comprehensively understand the complex interplay of factors affecting dental service utilization and edentulism patterns across different populations.

The study conducted by Yuan L. et.al. [22] found that older individuals living in urban areas in China were more likely to use dentures compared to those in rural areas, with a 67.05% denture use rate in urban participants compared to 51.12% in rural participants. The gap in denture use was influenced by several factors, including education level, exercise habits, and chronic diseases, with urban residents benefiting from better access to dental services and higher socioeconomic status. In contrast, rural residents faced challenges such as fewer educational opportunities, limited access to dental care, and higher rates of chronic health conditions, which contributed to the disparity in denture use.

There is a clear connection between the type of edentulism and risk factors like alcoholism and smoking [7,8,9,10,11,12]. Both behaviors greatly contribute to tooth loss by negatively impacting oral health, such as increasing the risk of periodontal disease and weakening the body’s ability to fight infections. The current study demonstrated that patients who consume alcohol or smoke show a higher prevalence of class 1 and total edentulism.

Similarly, a study by Duarte PM et al. [23] found that smokers are more likely to develop class I and total edentulism compared to non-smokers, particularly in the posterior region of the dental arch. The study emphasized that smokers are more likely to experience gum recession and bone loss, which leads to early tooth loss. Smoking also reduces blood flow to the gums, impairing healing after dental procedures and accelerating the progression of periodontal disease, further contributing to edentulism. Excessive alcohol consumption is another major risk factor for tooth loss. A study by Oliveira et al. [24] found that heavy alcohol consumption is strongly linked to higher rates of tooth loss, particularly among older adults with lower socioeconomic status. The research showed that heavy alcohol users, especially those from low-wealth households or with lower education levels, had a significantly higher prevalence of tooth loss. The study highlighted that the risk of tooth loss was compounded by socioeconomic disadvantages, with individuals facing both low income and education being more likely to suffer from complete edentulism. As with smoking, the frequency and duration of alcohol use were critical factors in determining the severity of tooth loss, with chronic alcohol users being at a notably higher risk of edentulism later in life.

The current study presents several limitations. The first is its cross-sectional design, as it offers only a single-time-point snapshot of the relationship, which might not be applicable to other populations or timeframes. A longitudinal design could help track individual changes in edentulism status over time, enabling the identification of potential predictors with more confidence. Additionally, the reliance on self-reported questionnaires introduces the possibility of recall bias or social desirability bias, which may affect the accuracy and reliability of the responses. Furthermore, various confounding factors, such as dietary habits, smoking, oral hygiene practices, and genetic predisposition, may influence both edentulism and general health, but were not fully controlled for in this study. Another limitation pertains to the sample size; although it fell within the calculated optimal range, it was at the lower end of the interval. A larger sample size would have enhanced the statistical power of this study, increasing the ability to detect subtle yet meaningful associations between variables. Additionally, using objective measures of health and dental status would reduce reliance on self-reported data and minimize bias. Including more diverse populations would improve this study’s statistical power and generalizability. Investigating risk factors indicating the association of frequency and length of alcohol and smoking with edentulism, as well as potential confounders, such as genetic factors and specific health behaviors, could offer deeper insights into the influences on edentulism and general health.

## 5. Conclusions

The severity of edentulism increases with age, particularly with a shift toward class 1 and complete tooth loss in older individuals. Furthermore, edentulism is strongly associated with overall health, influencing nutritional intake and increasing the risk of systemic diseases such as cardiovascular disease and diabetes. Risk factors like smoking and excessive alcohol consumption also play a major role in accelerating tooth loss, with smokers and heavy drinkers experiencing higher rates of class I and total edentulism.

## Figures and Tables

**Table 1:** Correlation between gender and type of edentulism in the maxilla.

	Maxillary Edentulous Class					Point
	All Teeth	Cls 1	Cls 2	Cls 3	No Teeth	*p*
Female	14 (11%)	25 (19.7%)	22 (17.3%)	33 (26%)	33 (26%)	0.339
Male	5 (6.2%)	13 (16%)	10 (12.3%)	30 (37%)	23 (28.4%)	
Total	19 (9.1%)	38 (18.3%)	32 (15.4%)	63 (30.3%)	56 (26.9%)	

**Table 2:** Correlation between gender and type of edentulism in the mandible.

	Mandibular Edentulous Class					Point
	All Teeth	Cls 1	Cls 2	Cls 3	No Teeth	*p*
Female	9 (7.1%)	46 (36.2%)	16 (12.6%)	38 (29.9%)	18 (14.2%)	0.321
Male	9 (11.1%)	22 (27.2%)	12 (14.8%)	20 (24.7%)	18 (22.2%)	
Total	18 (8.7%)	68 (32.7%)	28 (13.5%)	58 (27.9%)	36 (17.3%)	

**Table 3:** Distribution of patients according to sex and age.

Age			
	20–40 (*n*, %)	41–59 (*n*, %)	≥60 (*n*, %)
Female	35 (27.6)	45 (35.4)	47 (37)
Male	23 (28.4)	27 (33.3)	31 (38.8)

**Table 4:** Correlation between general health and type of edentulism in the maxilla.

	Maxillary Edentulous Class						
Disease	All Teeth (*n*, %)	Cls 1 (*n*, %)	Cls 2 (*n*, %)	Cls 3 (*n*, %)	No Teeth (*n*, %)	Total (*n*, %)	*p*
Cardiovascular	3 (27.27)	14 (35)	10 (2.94)	19 (35.19)	27 (39.13)	73 (35.10)	0.082
Digestive	0	4 (10)	4 (11.46)	5 (9.26)	11 (15.94)	24 (11.54)	0.139
Diabetes	3 (27.27)	7 (17.5)	11 (32.35)	6 (11.11)	8 (11.59)	35 (16.83)	0.475
Neurological	1 (9.09)	4 (10)	0	3 (5.56)	6 (8.70)	14 (6.73)	0.282
Osteoarticular	1 (9.09)	4 (10)	4 (11.46)	3 (5.56)	8 (11.59)	20 (9.62)	0.424
Generally Healthy	3 (27.27)	7 (17.50)	5 (14.71)	18 (33.33)	9 (3.04)	42 (20.19)	
Total	11 (100)	40 (100)	34 (100)	54 (100)	69 (100)	208 (100)	

**Table 5:** Correlation between general health and type of edentulism in the mandible.

	Mandibular Edentulous Class						Point
Disease	All Teeth (*n*, %)	Cls 1 (*n*, %)	Cls 2 (*n*, %)	Cls 3 (*n*, %)	No Teeth (*n*, %)	Total (*n*, %)	*p*
Cardiovascular	8 (38.10)	18 (26.87)	11 (44)	15 (30.61)	21 (45.65)	73 (35.10)	0.008
Digestive	2 (9.52)	7 (10.45)	4 (16)	6 (12.24)	5 (10.87)	24 (11.54)	0.964
Diabetes	3 (14.28)	14 (20.89)	5 (20)	8 (16.32)	5 (10.87)	35 (16.83)	0.839
Neurological	1 (4.76)	4 (5.97)	0	5 (10.20)	4 (8.70)	14 (6.73)	0.468
Osteoarticular	1 (4.76)	10 (14.93)	1 (4)	2 (4.08)	6 (13.04)	20 (9.62)	0.086
Generally Healthy	6 (28.57)	14 (20.90)	4 (16)	13 (26.53)	5 (10.87)	42 (20.19)	
Total	21 (100)	67 (100)	25 (100)	49 (100)	46 (100)	208 (100)	

## Data Availability

Data available in a publicly accessible repository.

## References

[1] Park H.A., Shin S.H., Ryu J.I. (2023). Edentulous disparities among geriatric population according to the sexual difference in South Korea: A nationwide population-based study. Sci. Rep..

[2] Van der Putten G.J. (2019). De relatie mondgezondheid en algemene gezondheid bij ouderen [The relationship between oral health and general health in the elderly]. Ned. Tijdschr. Tandheelkd..

[3] Kheir O.O., Ziada H.M., Abubakr N.H., Abdel-Rahman M.E., Fadl S.M., Ibrahim Y.E. (2019). Patient-dentist relationship and dental anxiety among young Sudanese adult patients. Int. Dent. J..

[4] Al-Rafee M.A. (2020). The epidemiology of edentulism and the associated factors: A literature Review. J. Fam. Med. Prim. Care.

[5] Celeste R.K., Darin-Mattsson A., Lennartsson C., Listl S., Peres M.A., Fritzell J. (2021). Social Mobility and Tooth Loss: A Systematic Review and Meta-analysis. J. Dent. Res..

[6] Kassebaum N.J., Smith A.G.C., Bernabé E., Fleming T.D., Reynolds A.E., Vos T., Murray C.J.L., Marcenes W., GBD 2015 Oral Health Collaborators (2017). Global, Regional, and National Prevalence, Incidence, and Disability-Adjusted Life Years for Oral Conditions for 195 Countries, 1990–2015: A Systematic Analysis for the Global Burden of Diseases, Injuries, and Risk Factors. J. Dent. Res..

[7] Edlibi Al Hage W., Dascălu C.G., Balcoș C., Agop-Forna D., Forna N.C. (2022). Trends in Access to Oral Health Care among Adults from the N-E Region of Romania. Medicina.

[8] Kovács N., Liska O., Idara-Umoren E.O., Mahrouseh N., Varga O. (2023). Trends in dental care utilisation among the elderly using longitudinal data from 14 European countries: A multilevel analysis. PLoS ONE.

[9] Janto M., Iurcov R., Moca A.E., Daina C.M., Moca R.T., Daina L.G. (2023). The Epidemiology of Dental Pathologies in Elderly Patients Admitted to a Tertiary Level Hospital in Oradea, NW Romania: A 5-Year Retrospective Study. Healthcare.

[10] Iosif L., Preoteasa C.T., Preoteasa E., Ispas A., Ilinca R., Murariu-Mǎgureanu C., Amza O.E. (2021). Oral health related quality of life and prosthetic status among institutionalized elderly from the bucharest area: A pilot study. Int. J. Environ. Res. Public Health.

[11] Baumeister S.E., Freuer D., Nolde M., Kocher T., Baurecht H., Khazaei Y., Ehmke B., Holtfreter B. (2021). Testing the association between tobacco smoking, alcohol consumption, and risk of periodontitis: A Mendelian randomization study. J. Clin. Periodontol..

[12] Jing T., Vaithilingam R.D. (2020). Alcohol consumption is associated with periodontitis. A systematic review and meta-analysis of observational studies. Community Dent. Health.

[13] Galindo-Moreno P., Lopez-Chaichio L., Padial-Molina M., Avila-Ortiz G., O’Valle F., Ravida A., Catena A. (2022). The impact of tooth loss on cognitive function. Clin. Oral Investig..

[14] Khurshid Z., Waqas M., Hasan S., Kazmi S., Faheemuddin M. (2025). Deep Learning Architecture to Infer Kennedy Classification of Partially Edentulous Arches Using Object Detection Techniques and Piecewise Annotations. Int. Dent. J..

[15] Rajeh M.T. (2022). Gender Differences in Oral Health Knowledge and Practices Among Adults in Jeddah, Saudi Arabia. Clin. Cosmet. Investig. Dent..

[16] Jeyapalan V., Krishnan C.S. (2015). Partial Edentulism and its Correlation to Age, Gender, Socio-economic Status and Incidence of Various Kennedy’s Classes—A Literature Review. J. Clin. Diagn. Res..

[17] Almusallam S.M., Alrafee M.A. (2020). The prevalence of partial edentulism and complete edentulism among adults and above population of Riyadh city in Saudi Arabia. J. Fam. Med. Prim. Care.

[18] Zaigham A.M., Muneer M.U. (2010). Pattern of partial edentulism and its association with age and gender. Pak. Oral Dent. J..

[19] Kaurani P., Kakodkar P., Bhowmick A., Samra R.K., Bansal V. (2024). Association of tooth loss and nutritional status in adults: An overview of systematic reviews. BMC Oral Health.

[20] Chapple I.L., Genco R., Working Group 2 of the Joint EFP/AAP Workshop (2013). Diabetes and periodontal diseases: Consensus report of the Joint EFP/AAP Workshop on Periodontitis and Systemic Diseases. J. Periodontol..

[21] Luo H., Wu Q., Bell R.A., Wright W., Quandt S.A., Basu R., Moss M.E. (2021). Rural-Urban differences in dental service utilization and dental service procedures received among US adults: Results from the 2016 medical expenditure panel survey. J. Rural. Health.

[22] Yuan L., Yuan Y., Ren H., Zhang F., Zhao Z., Jiang Q., Wei Z., Sun J.H. (2024). Decomposition analysis of the prevalence of denture use between rural and urban older individuals with edentulism in China: A Cross-Sectional study. Interact. J. Med. Res..

[23] Duarte P.M., Nogueira C.F.P., Silva S.M., Pannuti C.M., Schey K.C., Miranda T.S. (2022). Impact of Smoking Cessation on Periodontal Tissues. Int. Dent. J..

[24] Oliveira L.M., Pelissari T.R., Moreira C.H.C., Ardenghi T.M., Demarco F.F., Zanatta F.B. (2023). The alcohol harm paradox and tooth loss among Brazilian older adults. Oral Dis..

